# Effects of a single application of hydrolyzed fish collagen on dermal protein expression and tissue architecture in human skin models

**DOI:** 10.1038/s41598-025-11372-5

**Published:** 2025-08-11

**Authors:** Lorenzo Dondero, Giulia De Negri Atanasio, Erica Lertora, Francesca Tardanico, Ilaria Demori, Pietro Storace, Giorgia Allaria, Francesca Rispo, Federica Robino, Elisabetta Perata, Matteo Zanotti-Russo, Sara Ferrando, Elena Grasselli

**Affiliations:** 1 Department of Earth, Environment and Life Science, University of Genoa, Genova, Italy; 2 Interuniversity Center for the Promotion of 3R Principles in Teaching and Research (Centro 3R), Pisa, Italy; 3 Angel Consulting, Via San Senatore 14, Milano, 20122 Italy; 4 Department of Pharmacy, University of Genoa, Viale Cembrano 4, Genova, 16148 Italy; 5 National Biodiversity Future Center Piazza Marina, Palermo, 61 90133 Italy; 6 National Center for the Development of New Technologies in Agriculture (Agritech), Napoli, 80121 Italy

**Keywords:** Hydrolyzed fish collagen, Circular economy, Skin ageing, New approach methodologies, Next generation risk assessment, Biotechnology, Cell biology, Molecular biology

## Abstract

The use of collagen as an active ingredient in cosmetic products is becoming increasingly common. Recently, scientific interest has been focused on fish collagen, which is similar to human collagen and can be extracted from fish by-products. This study evaluates the cosmetic potential of Hydrolyzed Fish Collagen (HFC), testing it both as an aqueous solution (S-HFC) and as a formulation in cosmetic products (F-HFC) containing the same amount of HFC. The effects of S-HFC and F-HFC were evaluated on 2D (endothelial and keratinocyte cells) and 3D (EpiDerm and EpiDerm-FT) skin models. The results demonstrated that HFC, as an active ingredient, shows promising effects on skin physiology, including increased cell viability, enhanced wound healing, and an anti-wrinkle effect.

**Supplementary Information:**

The online version contains supplementary material available at 10.1038/s41598-025-11372-5.

## Introduction

Collagen is the predominant structural protein in the connective tissue of vertebrates, providing strength to the skin, bones, tendons, and cartilage^1^.

It’s known that 40 vertebrate genes make up the entire collagen family that is composed of 29 different molecules grouped into 8 families depending on structure, chain-bonding and position in the human body^2^.

Generally, the primary composition of collagen predominantly comprises the amino acids glycine (Gly) (33%), proline (Pro) (22%), and hydroxyproline (Hyp) (14%)^3^. In particular, the Hyp content is about one sixth of the total collagen mass. Moreover, this post-translationally hydroxylated amino acid, that is found massively in collagen, is present to a much lesser extent in other proteins (e.g. some kinases)^4^; for these reasons, it stands for the representative amino acid of the collagen molecule^5^.

The typical collagen structure forms a triple helix, constituted by three α-chains. Each α-chain is comprised of approximately 1024 amino acids, resulting in a molecular weight of approximately 105 kDa^6,7^.

Indeed, collagen stands as the principal structural protein in both the extracellular matrix (ECM) and the connective tissue of animals. Among mammals, collagen is notably abundant, predominantly situated in the ECM of fibrous connective tissues, including tendons and skin^8^. It plays key structural roles by supporting the formation, tensile strength, flexibility of joints, cell migration, and differentiation functions^9^. In particular, it is known that 80–85% of the dermal ECM is composed by collagen type I and collagen type III (8–11%)^10^.

Since skin acts as a barrier to protect the human body from dehydration, penetration of various microorganisms, allergens, irritants, reactive oxygen species and radiation, daily skin care is important to maintain skin regeneration, elasticity and smoothness. The human skin is continuously deteriorated by different factors, which can influence and accelerate skin ageing (environmental conditions, smoking, alcohol, stress, sun exposure, etc.)^11^. On the other hand, studies have provided evidence that collagen synthesis undergoes alterations across the various life stages, notably affecting the relative proportions of collagen types within the skin. As individuals age, there is a natural decline in the ability to replenish collagen, diminished by approximately 1.5% annually. Over time, collagen fibers in mature skin become thicker and much shorter, leading to a reduction in type I collagen content, thereby altering the balance of collagen types. Concurrently, the density of both collagen and elastin in the dermis diminishes, resulting in a decline in the structural integrity and elasticity of the skin. This transformation leads to a thinner and less pliable skin texture^12^.

To counteract skin ageing, the use of collagen as an active ingredient in cosmetic products is promising for its moisturizing, regenerating and film-forming properties^13^. Hydrolyzed collagen (HC), composed by small and short peptides (molecular weight < 3 kDa) derived from the entire collagen molecule are usually included in cosmetic formulations, because they are easily dissolved in water at neutral pH^14^. Most importantly, the low molecular weight of HC allows it to penetrate the deepest layers of the skin, where it directly contributes to maintaining and improving skin hydration^15^. This is due to the presence of several Natural Moisturizing Factors (NMFs), such as Serine, Aspartic Acid, Hydroxylysine, Hydroxyproline, and others^16^. Moreover, HC has been extensively documented to possess antioxidant properties, thus allowing to counteract skin ageing triggered by oxidative stress^17^. Another ability of collagen and HC is to enhance wound healing by promoting tissue regeneration^18^; this property is essential to maintain the integrity of the skin that act as a major protective barrier of the human body^19^.

Collagen and collagen derivatives can be obtained from various sources, including vertebrates and invertebrates. Common collagen sources are porcine and bovine skin and bones, but recently fish collagen, extracted from sources such as fish and other aquatic organisms, has gained scientific interest and represents an interesting alternative to mammalian commercial sources that can be associated with zoonotic diseases (e.g. transmissible spongiform encephalopathy, bovine spongiform encephalopathy, foot and mouth disease)^14^. Moreover, fish collagen is remarkably similar to human collagen, thus showing potential applications in regenerative medicine, cosmetics, and food industry^20^. Fish collagen demonstrates excellent skin absorption and contributes to anti-aging, hydrating, and skin-repairing effects, making it well-suited for cosmetic formulations^21^.

Fish-derived collagen is often obtained from low-value or discarded materials from the seafood industry, making it a more environmentally sustainable choice. Unlike bovine or porcine collagen, it avoids concerns related to ethics, religion, and health, which broadens its appeal across a wide range of consumers. Due to these advantages, marine collagen is increasingly seen as a preferable ingredient in cosmetic formulations, especially in response to the rising interest in clean-label, ethical, and high-performance skincare products^22^.

In this article, we will delve into the biochemical and physiological characteristics of a Hydrolyzed Fish Collagen (HFC) derived from Tilapia and Pangasius skin. This specific HFC, characterized by an average molecular weight of approximately 2 kDa and a protein content of 90%, is not an experimental prototype but rather a benchmark product already widely used as a cosmetic ingredient in commercial formulations. Its inclusion in marketed products underscores both its safety and functional relevance in skin care applications. The Tilapia and Pangasius species were chosen for collagen production primarily due to their high availability, low cost, and the large amount of by-products generated during their processing. These fish, widely consumed in their countries of origin, are farmed in large quantities, making them a cost-effective option for non-food applications like collagen production. Although not native to the Mediterranean, their low price and global availability make them a practical choice^23,24^.

Given the rising popularity of fish collagen in cosmetic and dermatological products, particularly those with anti-aging or skin-repair claims, we chose to investigate HFC using human skin models. We will examine the implications for cosmetic use, and we will compare an aqueous solution of HFC (S-HFC) to a commercial face cream formulated with the same amount of HFC as active ingredient (F-HFC). Cell viability and skin irritation test, wound healing, anti-wrinkle effects, hydration, and elasticity will be evaluated in 2D and 3D in vitro models. Despite its established use in marketed formulations, to the best of our knowledge, the safety and efficacy of HFC have not yet been systematically investigated using advanced 3D skin models. Therefore, there is a clear need to evaluate its biological behavior in such systems, in order to provide robust and mechanistically grounded evidence supporting its cosmetic relevance.

## Results

### Effect of S-HFC on viability and wound healing of human keratinocytes and endotheliocytes

As preliminary assessment of the quality of the collagen used for the following tests, we performed Ultra-Violet (UV) spectroscopy analysis and evaluation of Hyp content. The UV spectrum analysis of HFC revealed a discernible absorption peak at 228 nm (Abs 1.680), indicative of the presence of peptide bonds, and a minor peak around 231 nm (Abs 0.866) (Supplementary data, Figure S1). It is noteworthy the absence of the peak at 280 nm, due to the absence of aromatic residues in the collagen sequence. On the other hand, the Hyp content was evaluated on HFC sample: a concentration of 47.007 ± 1.36 µg_Hyp_/mL was detected. We chose to indicate the amount of Hyp for the further treatments to both cells and tissues.

**Fig. 1:**
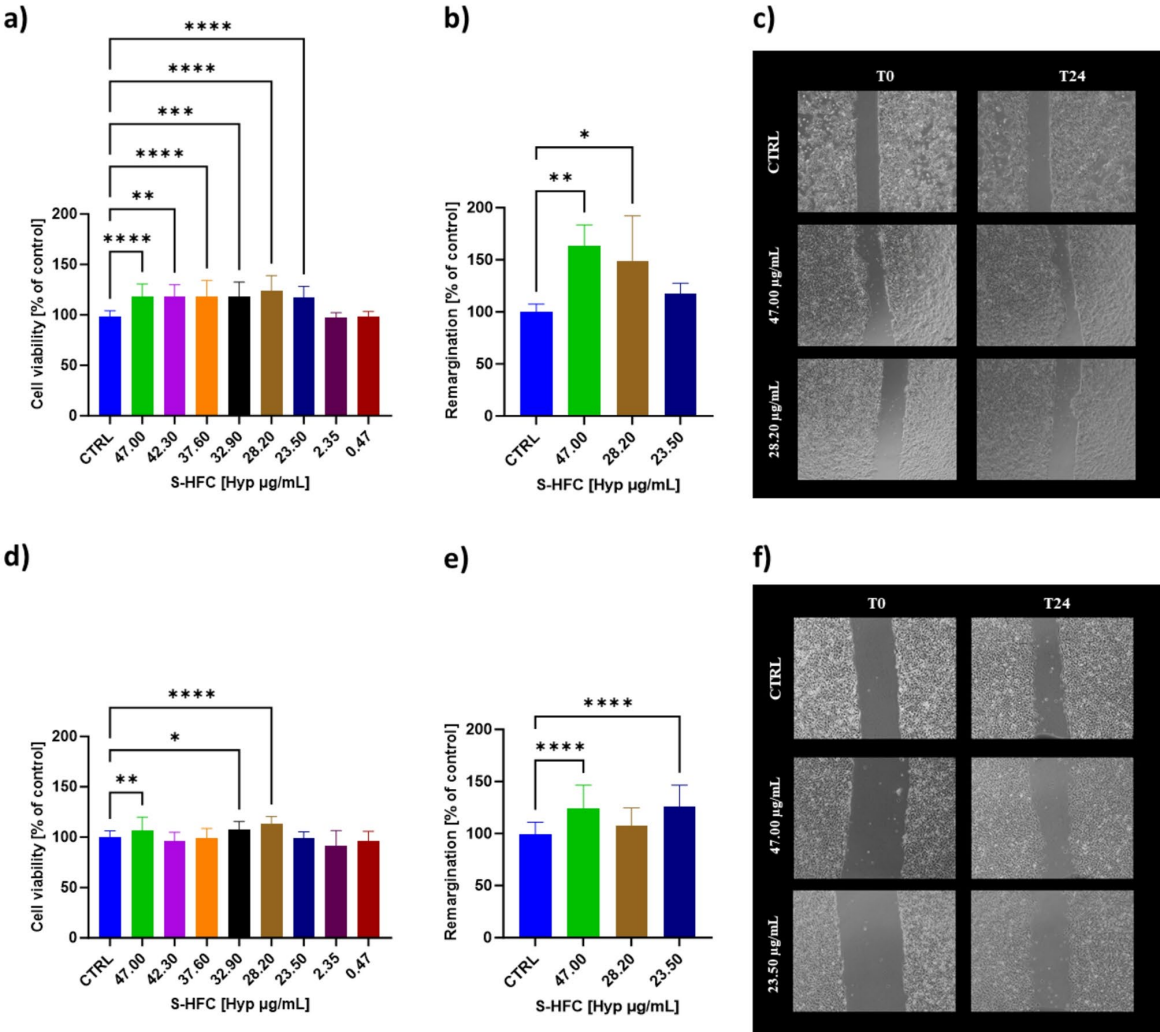
Cell viability, wound healing percentage and representative images of HaCaT (**a**,** b**,** c**) and HECV (**d**,** e**,** f**) cells, treated for 24 h with S-HFC at different concentrations (47.00; 28.20; 23.50 µg_Hyp_/mL, corresponding to 0.1%; 0.06% and 0.05% w/v of HFC, respectively). Data are mean ± SD from at least 3 independent experiments performed in triplicate and are expressed as percentage of CTRL (vehicle alone). The significant differences are indicated by symbols on bars (**** *p* ≤ 0.0001, *** *p* ≤ 0.001, ** *p* ≤ 0.01, *p ≤ 0.05 vs. CTRL).

Effects on cell viability was measured as a function of S-HFC treatment at different concentrations (expressed as Hyp content µg_Hyp_/mL) in both Human Keratinocytes (HaCaT) and Human endothelial-derived from umbilical cord vein (HECV) cells. After 24 h of treatment, S-HFC was able to stimulate cell viability in both cellular models analyzed. HaCaT cells were stimulated in terms of cell viability from 47.00 to 23.50 µg_Hyp_/mL. The effects of S-HFC treatment on HECV cells showed a different trend since the stimulation of cell viability was detected at 47.00, 32.90 and 28.20 µg_Hyp_/mL, thus indicating a non-monotonic dose-response curve. No significant differences were observed for the cell lines treated with all the other concentrations, as opposed to the negative control (CTRL) (Fig. 1a, d). Moreover, T-scratch test was performed as a simple assay aimed at evaluating the stimulation of wound healing by S-HFC in a 2D cellular system. Wound healing (WH) resulted increased for both HaCaT and HECV cells after treatment with S-HFC for 24 h at 47.00 µg_Hyp_/mL, whereas in HaCaT cells WH was stimulated also by 28.20 µg_Hyp_/mL and in HECV by 23.50 µg_Hyp_/mL (Fig. 1b, c, e, f).

### Effect of F-HFC on cell viability of keratinocyte cell cultures

**Fig. 2:**
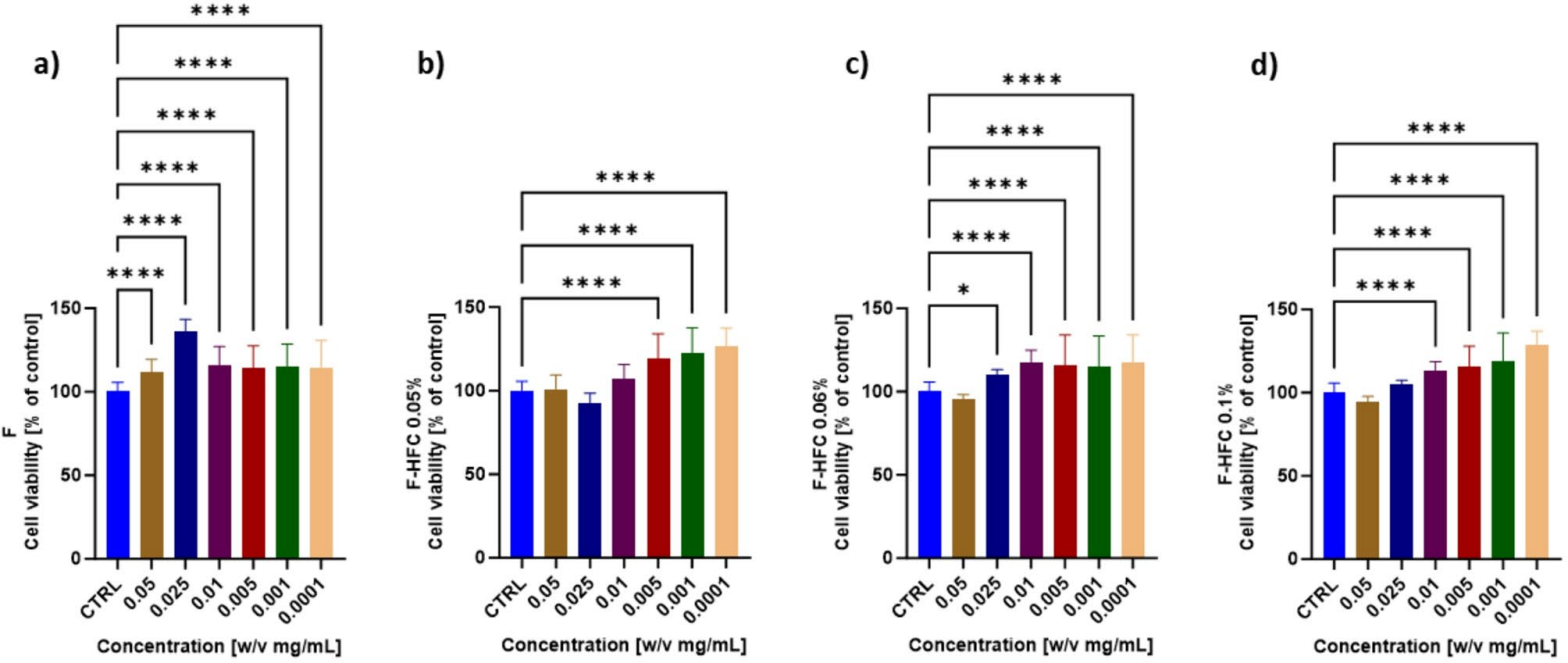
Cell viability of HaCaT cells treated for 24 h with: (**a**) a commercial face cream (F); (**b**,** c**,** d**) F supplemented with HFC (F-HFC) at different concentrations (0.05%; 0.06% and 0.1% w/v, corresponding to 23.50; 28.20; 47.00 µg_Hyp_/mL, respectively). F-HFC was tested at different dilutions in culture medium (w/v: from 0.05 to 0.0001 mg/mL). The results are expressed as percentage of control (CTRL, vehicle alone). Values are mean ± SD from at least 3 independent experiments performed in triplicates. The significant differences are indicated by symbols on bars (**** *p* ≤ 0.0001, * *p* ≤ 0.05 vs. CTRL).

F-HFC was formulated by adding HFC at different percentages (0.05%; 0.06% and 0.1%, corresponding to 23.50 µg_Hyp_/mL, 28.20 µg_Hyp_/mL, and 47.00 µg_Hyp_/mL) to a commercial face cream; the concentrations were chosen based on their effectiveness in stimulating both cellular viability and WH in HaCaT cells (Fig. 1). Each formulation was diluted in D-MEM at different w/v concentrations and cells were treated for 24 h. The cells were also exposed to the commercial face cream without addition of HFC (F). Since the cream is applied to the skin surface, we aimed to evaluate F and F-HFC effects on the viability of keratinocytes, in particular. Figure 2 shows the viability of HaCaT cells as a function of the different treatments. Compared to vehicle alone (CTRL), F significantly enhanced cell viability across all concentrations tested, whereas F-HFC was effective for concentrations ranging from 0.025 to 0.0001 mg/mL. However, no significant differences were observed in the extent of cell viability increase across all conditions tested.

### Effects of S-HFC and F-HFC on EpiDerm 3D skin model

**Fig. 3:**
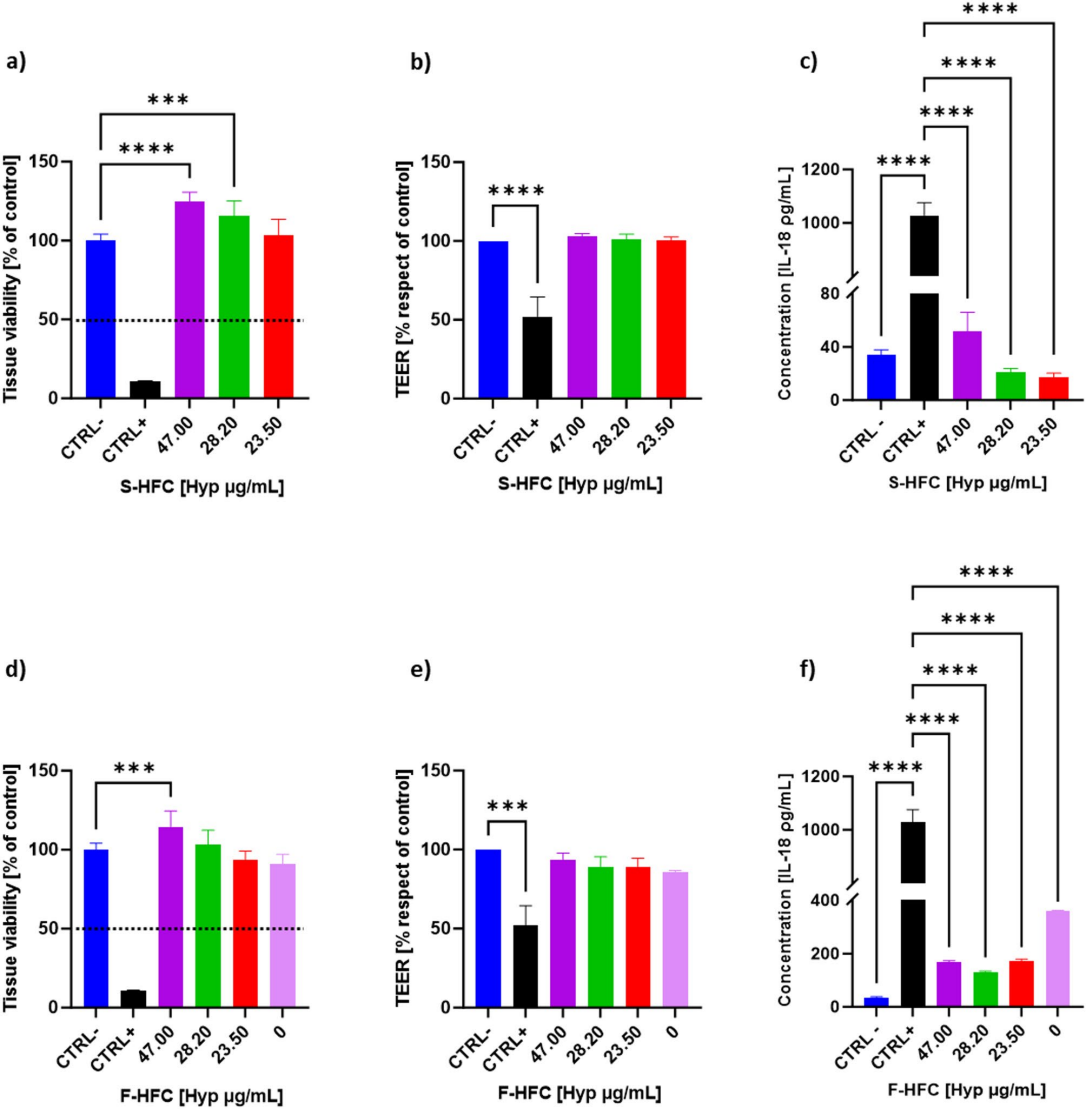
Tissue viability, TEER measurement and IL-18 secretion in 3D EpiDerm models treated with S-HFC (**a**,** b**,** c**) and F-HFC (**d**,** e**,** f**) at different concentrations (47.00; 28.20; 23.50 µg/mL, corresponding to 0.1%, 0.06% and 0.05% w/v of HFC) for 60 ± 1 min, compared to negative controls (CTRL-, vehicle alone). Positive controls (CTRL+) were treated with 5% SDS, whereas 0.00 µg_Hyp_/mL stands for F (cream without addition of HFC). Values are mean ± SD from at least 3 independent experiments performed in triplicate. The significant differences are indicated by symbols on bars (**** *p* ≤ 0.0001, *** *p* ≤ 0.001, **p ≤ 0.01 vs. CTRL-).

The EpiDerm 3D skin in vitro model was used to evaluate the safety of S-HFC and F-HFC through the skin irritation test, according to OECD 439 guidelines. The test consists in identifying an irritating cosmetic product by its ability to decrease cell viability below defined threshold levels (i.e. ≤ 50% for UN GHS Category 2)^25^. Figure 3 (a; d) shows the viability of the epidermal tissue after 1 h of treatment with S-HFC (47.00, 28.20, 23.50 µg_Hyp_/mL) and F-HFC (47.00, 28.20, 23.50 and 0.00 µg_Hyp_/mL). According to the OECD guideline 439, the tissue viability upon treatment with S-HFC and F-HFC was not altered, even resulting in a statistically significant increase in the presence of 47.00 µg_Hyp_/mL for both S-HFC and F-HFC and of 28.20 µg_Hyp_/mL for S-HFC only, as compared to the negative control (CTRL-). Thus, both S-HFC and F-HFC can be considered safe and non-irritant to the skin across all concentrations tested.

Transepithelial Electrical Resistance (TEER) was measured on the same tissues that underwent the skin irritation test. As shown in Fig. 3b and e, no differences were detected in tissues treated with both S-HFC and F-HFC respect to vehicle alone (CTRL-), indicating the maintenance of the tissue barrier function across all treatments tested.

The secretion of interleukin-18 (IL-18) in the culture media was assessed. As shown in Fig. 3c, f and S-HFC treatments did not significantly increase IL-18 secretion during the incubation period (60 ± 1 min). On the other hand, F-HFC treatments significantly enhanced IL-18 secretion, but to a significantly lesser extent with respect to the positive control (CTRL + is 5% SDS). Overall, the results suggest the absence of an ongoing inflammatory state in the tissues treated with both S-HFC and F-HFC for all concentrations tested.

### Anti-wrinkle effect of S-HFC and F-HFC on a reconstructed 3D skin model

To investigate the anti-wrinkle properties of both S-HFC and F-HFC, we assessed their effects on gene expression and tissue histology (collagen deposition and fibroblast count within the dermis) using an additional 3D skin model: the EpiDerm FT (Full Thickness) model, which includes both epidermis and dermis layers. To evaluate the stimulation of collagen production, we measured the expression of genes encoding for collagen isoforms (Collagen Type III Alpha 1 Chain (COL3A1) and Collagen Type I Alpha 1 Chain (COL1A1)). Moreover, collagen deposition was evaluated by Sirius Red (SR) staining and by counting the number of fibroblasts within the dermis stratum.

**Fig. 4:**
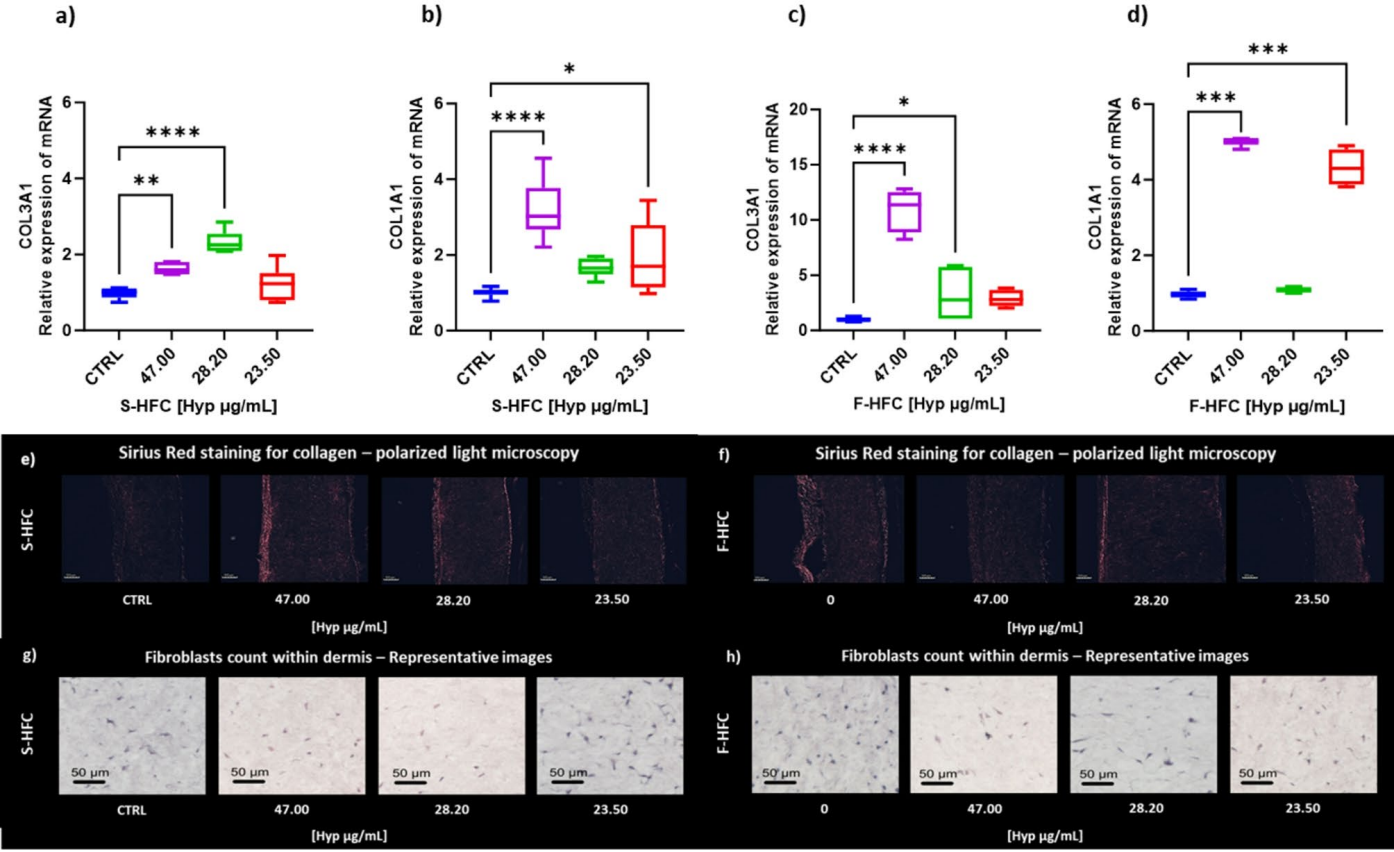
Relative gene expression of Collagen Type III Alpha 1 Chain (COL3A1) and Collagen Type I Alpha 1 Chain (COL1A1), Collagen staining, and Fibroblasts count evaluated in 3D EpiDerm FT models treated for 24 h with S-HFC (**a**,** b**,** e**,** g**) and F-HFC (**c**,** d**,** f**,** h**) respectively, at different concentrations (47.00; 28.20; 23.50 µg_Hyp_/mL, corresponding to 0.1%; 0.06% and 0.05% w/v of HFC), with respect to controls (CTRL for S-HFC, consisting in vehicle, and 0.00 µg_Hyp_/mL for F-HFC consisting in the cream without addition of HFC). In the box-and-whisker plots the horizontal line indicates the median, the box reflects values from the first to the third quartile, and the whiskers represent the furthest points that are not outliers. Values come from at least 2 independent experiments performed in triplicate. The significant differences are indicated by symbols on bars (**** *p* ≤ 0.0001, *** *p* ≤ 0.001, ** *p* ≤ 0.01, * *p* ≤ 0.05 vs. CTRL).

Figure 4 shows the gene expression of COL3A1 and COL1A1 following a 24-hour treatment with S-HFC (47.00; 28.20; 23.50 µg_Hyp_/mL) and F-HFC (47.00; 28.20; 23.50; 0.00 µg_Hyp_/mL).

COL3A1 mRNA expression was induced at the highest concentrations of S-HFC, while the expression of COL1A1 was induced at both 47.00 µg_Hyp_/mL and 23.50 µg_Hyp_/mL. Similar results were observed for F-HFC, but at a higher order of magnitude: the median effect of F-HFC at 47.00 µg_Hyp_/mL on COL3A1 mRNA levels was 7 times bigger than the median effect of S-HFC at the same concentration (11.37- vs. 1.59-fold induction). These results indicate a higher efficacy of F-HFC compared to S-HFC. Moreover, the increases in COL3A1 and COL1A1 mRNA levels by S-HFC and F-HFC were confirmed by SR collagen staining.

As shown in Fig. 4e and f, the amount of collagen stained with SR was more evident upon treatment with 47.00 µg_Hyp_/mL for both S-HFC and F-HFC. On the contrary, the number of fibroblasts cells within the dermis did not differ significantly across all conditions tested (Fig. 4g and h).

### Effects of S-HFC and F-HFC on hydration and elasticity on a reconstructed 3D skin model

**Fig. 5:**
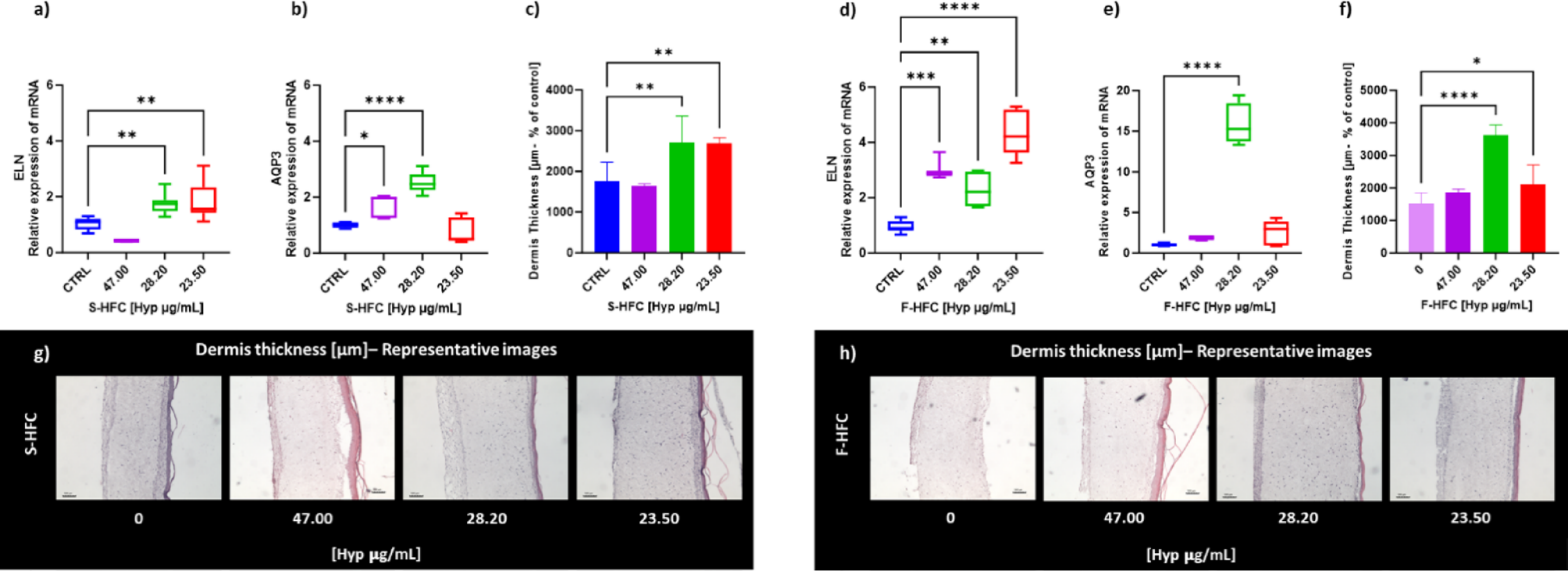
Relative gene expression of Elastin (ELN) and Aquaporin-3 (AQP3), Dermis thickness measurement, and representative images in 3D EpiDerm FT models treated for 24 h with S-HFC (a, b, c, g) and F-HFC (d, e, f, h) respectively, at different concentrations (47.00; 28.20; 23.50 µg_Hyp_/mL, corresponding to 0.1%; 0.06% and 0.05% w/v of HFC), with respect to negative controls (CTRL for S-HFC, consisting in vehicle, and 0.00 µg_Hyp_/mL for F-HFC, consisting in the cream without addition of HFC). For each treatment, dermis thickness was measured cutting five different hematoxylin-eosin stained sections of 5 μm thickness. In the box-and-whisker plots, the horizontal line indicates the median, the box reflects values from the first to the third quartile, and the whiskers represent the furthest points that are not outliers. Values come from at least 2 independent experiments performed in triplicate. The significant differences are indicated by symbols on bars (**** *p* ≤ 0.0001, *** *p* ≤ 0.001, ** *p* ≤ 0.01, * *p* ≤ 0.05 vs. CTRL).

Gene expression analysis of AQP3 (Aquaporin-3) and ELN (Elastin), and dermis thickness measurement in the 3D EpiDerm FT models were used to evaluate elasticity and hydration following a 24-hour treatment with S-HFC or F-HFC. S-HFC induced ELN expression at the lowest concentration tested, while AQP3 mRNA levels were also significantly increased at 28.20 µg_Hyp_/mL (Fig. 5a, b). As shown in Fig. 5d and e, F-HFC at 28.20 µg_Hyp_/mL was able to enhance AQP3 gene expression with a median 15.27-fold induction respect to a 2.47-fold induction for S-HFC. Additionally, ELN mRNA levels were increased across all tested Hyp concentrations, with particular emphasis on F-HFC at 23.50 µg_Hyp_/mL.

It is known that while the young dermis has a well-organized tight matrix, in aged skin the loss of matrix proteins occurs, which results in a disorganized and loose matrix^26^. For this reason, high levels of skin hydration are essential for proper skin barrier function, with particular emphasis on maintaining hydration balance in the extracellular matrix^27^. Thus, we randomly evaluated the variation of dermis thickness in the 3D EpiDerm FT skin models.

As shown in Fig. 5c, f, g, and h, the dermal thickness was increased by both S-HFC and F-HFC at the concentrations of 28.20 µg_Hyp_/mL and 23.50 µg_Hyp_/mL, respect to control.

## Discussion

This research investigates the cosmetic potential of hydrolyzed fish collagen (HFC, mean M.W. approximately 2 kDa), specifically derived from Tilapia and Pangasius skins, to promote skin regeneration and counteract skin aging. Collagen, a critical structural protein found in the skin, is primarily synthesized in the dermal layer. Its role is indispensable, providing vital support, firmness, and tensile strength to the skin. The stimulation of collagen production stands as a central objective in skincare research, as it can result in enhanced skin health and a noticeable reduction in visible signs of aging, such as wrinkles. Hydrolyzed collagen, with a low molecular weight, offers several advantages over native collagen, such as water solubility, cost-effectiveness, and sustainability, making it an ideal ingredient for cosmetic formulations^28^.

We chose to investigate HFC in human skin models because of its growing presence in cosmetic and dermatological products, especially those promoted for anti-aging or skin-repairing purposes. Despite its widespread use, there is still limited data on how HFC affects human skin at the cellular and molecular levels—particularly in terms of its impact on dermal protein expression (such as collagen, elastin, and aquaporin) and tissue morphology. Our study aims to fill this gap by providing experimental evidence on the effects of topical HFC using a biologically relevant skin model, helping to better define its efficacy and safety profile.

We compared an aqueous solution of HFC (S-HFC) to a commercial face cream formulated with the same amount of HFC as active ingredient (F-HFC). Preliminary observations identified the optimal concentrations of HFC for further testing. We decided to express such concentrations as the amount of Hyp (47.00, 28.20, 23.50 µgHyp/mL) rather than the w/v amount of collagen. Hyp is an amino acid distinctive to collagen proteins, and this makes it a direct and reliable marker for assessing the collagen content within the sample.

The tests on the HaCaT and HECV cell lines demonstrated that S-HFC promotes cell proliferation and accelerates wound closure in 2D cellular models. These results are in line with other findings documenting in both in vitro and in vivo experiments the wound healing activity of collagens extracted from *Chondrosia reniformis*^29^ and from *Rhopilema esculentum*^30^.

As a generalized proxy for cell and tissue status, we used the MTT assay. This method remains one of the most widely accepted and validated approaches for assessing cell viability in both 2D and 3D in vitro systems. In reconstructed 3D skin models, the MTT assay is a cornerstone of standardized toxicological evaluation, as outlined in OECD Test Guidelines 431^31^ and 439^32^, which rely on MTT reduction to assess tissue viability following chemical exposure. In this context, its use is essential to ensure compliance with international safety standards. In 2D monolayer cultures, the MTT assay provides a reliable measure of mitochondrial activity and general cell viability; however, these models inherently lack the tissue-level architecture and barrier functionality found in more complex systems, such as 3D reconstructed skin. Including it in our study enables consistent viability assessment across different model systems and provides a useful comparative benchmark for understanding how structural complexity influences cellular responses to the tested formulations. Accordingly, we employed 3D reconstructed epidermis (3D EpiDerm skin model). When applied to 3D skin models, both S-HFC and F-HFC were found to be safe and didn’t cause any signs of skin irritation at any concentration tested. Moreover, the tissue barrier function was maintained as indicated by TEER measurement, that provides an indicator of proper tight junction maintenance. On the other hand, we measured a slight but significant increase (with respect to negative control) of IL-18 secretion when tissues were treated with F-HFC. IL-18 is a pro-inflammatory cytokine involved in immune and inflammatory responses, and it is highly expressed in some skin diseases such as psoriasis, atopic dermatitis, drug allergy^33^. In any case, the IL-18 levels measured are not indicative of skin sensitization, as the measurement was carried out within the skin irritation protocol, which differs significantly in terms of exposure conditions and was not designed for this purpose^34^. It is also noteworthy that, when assessing the formulated compound, the IL-18 values recorded were lower than those observed for the formulation without HFC. This is likely due to the solvent effect, which can increase IL-18 secretion in a non-specific manner. The amount of IL-18 released under basal conditions significantly affects the ability to identify any reaction to the tested compound^35^. Importantly, this increase was not associated with any detrimental effects on tissue structure or function, as histological analysis (H&E staining) did not reveal any signs of inflammation or morphological disruption.

The increase in tissue viability observed for F-HFC was not mirrored by the same results in the 2D HaCaT model. This discrepancy may be attributed to the mandatory dilutions performed during the cellular treatment and/or the presence of a more complex mixture, which necessitates a more structured model.

Moreover, our study evaluated the effects of HFC on full-thickness 3D reconstructed epidermis, including both epidermal and dermal *strata*. The results revealed that HFC, both in soluble and formulated forms, enhanced collagen expression and deposition, particularly in the dermal matrix. This was associated with the upregulated expression of AQP3 and elastin expression, which could contribute to enhanced dermal structure suggesting that HFC can enhance skin hydration and may have anti-aging properties, contributing to wrinkle reduction and overall skin rejuvenation. The collagen deposition was evaluated using the Sirius Red (SR) staining method that is commonly used in histological analyses to highlight the presence of collagen in tissues. According to literature, SR binds specifically and primarily to fibrillar collagen^36,37^. Fibrillar collagen are a group of collagen proteins characterized by the formation of long, strong fibers with a typical triple-helical molecular structure. The main types of fibrillar collagen are type I, II, III, V and XI. Among these, Type I collagen constitutes approximately 90% of the total collagen in the human body, while type III collagen is mainly found in connective tissues^38^.

Of note, F-HFC significantly boosted its cosmetic efficacy, likely due to synergies with other natural active ingredients present in the commercial face cream, in addition to enhanced bioavailability^39^.In the era of New Approach Methodologies (NAMs), the 3D reconstructed skin models used in this study effectively mimic human skin, incorporating key cellular components and features, such as cellular crosstalk and compound permeability, which are characteristic of an intact organism. Compared to conventional 2D cultures, 3D models furnish a more precise depiction of how treatments impact collagen production. Moreover, these models align with the 3Rs approach, particularly focusing on replacement, as they represent physiologically relevant models that include the stratum corneum, epidermis, and dermis of human hairless skin. Since they are derived from human cells, these models do not exhibit biases that must be considered in animal testing. Additional reasons for using in vitro models include ethical considerations and legal requirements: in fact, according to Directive 2003/15/EC of the European Parliament and of the Council, the use of animals for testing cosmetic products or their ingredients is prohibited within the European Union.

Notably, 3D reconstructed skin models, produced under good manufacturing practices, are widely recognized as suitable tools for assessing the safety of products intended for human skin contact, such as cosmetics and medical devices^32^. To date, no standard operating procedures are available for testing the efficacy of a cosmetic product, and this aspect is entirely left to the scientific side of evidence from individual research groups. However, increased legislative demands for quality evidential claims point the attention to scientifically verify the cosmetic activity.

All experimental models present inherent limitations. In vivo animal models, for instance, are limited by species-specific differences that may not accurately reflect human physiology. On the other hand, clinical evaluations on human volunteers offer a more physiologically relevant scenario but often lack the ability for deep mechanistic analysis and are subject to individual variability and bias. In this light, 3D reconstructed skin models represent valuable tools for assessing the efficacy of cosmetic products^40^. These models enable controlled and reproducible testing conditions and are already widely accepted and incorporated into several internationally recognized standard operating procedures—particularly for safety assessments. Although no official standard operating procedures currently exist for efficacy testing, the use of 3D skin models represents a scientifically sound and increasingly adopted reference system in this field as well. Nonetheless, they can serve as a valuable complement to studies conducted on human volunteers, providing mechanistic insights under controlled conditions that support and strengthen clinical findings.

It is noteworthy that the concentrations of collagen we tested are relatively low, and our starting point was the existing literature on the biological effects of peptides. Several studies report that many peptides exhibit biological activity at very low, even parts-per-million (ppm) concentrations. This observation is consistent with our results and suggests the possibility of conducting additional assays to identify the optimal effective concentration for other active principles. Determining this concentration is important as it may be considerably lower than the amount used in the current formulation, potentially allowing for more efficient use of materials^41–43^. Moreover, with this research, we present, for the first time, a direct comparison of the efficacy of HFC as an isolated active principle (S-HFC) and within a commercial face cream (F-HFC), demonstrating that the formulation enhances the biological activity of HFC. Additionally, the study emphasizes the importance of using hydroxyproline (Hyp) as a more accurate indicator of collagen content and biological activity than the total mass of HFC, as variations in extraction methods and source species can affect the effective concentration.

We acknowledge some limitations inherent to the current study design. First, the absence of permeation kinetics data represents a constraint in defining the extent and timing of active compound delivery through the skin barrier. However, the aim of this study was not to model transdermal pharmacokinetics but to evaluate biological activity in a standardized in vitro environment where the compound is applied topically under conditions that mimic cosmetic use. In this context, the 3D reconstructed human skin model provides a physiologically relevant epidermal barrier, and is widely recognized as a suitable system for preliminary efficacy screening of topically applied compounds.

Second, the lack of quantification of HFC within the skin model may limit our ability to define precise dose–response relationships. Nonetheless, the experimental conditions were designed to reflect realistic application doses used in commercially available cosmetic formulations, and the significant biological responses observed suggest a relevant exposure level. Furthermore, the goal of this study was to assess functional endpoints—such as cell viability, barrier integrity, cytokine modulation, and gene expression—which can reveal mechanistic effects even in the absence of full quantitative absorption data.

While future studies incorporating compound quantification and permeation profiling (e.g., via mass spectrometry or radiolabeled tracers) will certainly enhance the translational relevance of the findings, the current approach provides a robust and reproducible platform for evaluating cosmetic efficacy. It offers valuable insight into the biological potential of HFC, supporting its further investigation under more complex or integrated models.

In conclusion, the study employs both 2D and 3D models to provide a comprehensive assessment of the biological activity of HFC and its formulated equivalent. Transitioning to the 3D models, we demonstrated the formulation’s ability to enhance the beneficial effects of the active principle. This is in line with the Next Generation Risk Assessment (NGRA) approach for the evaluation of both safety and efficacy of both active principle and cosmetic product^44^.

The results highlight the potential of HFC in cosmetic applications, particularly for skin regeneration and anti-aging. The application of HFC in cosmetic formulations also supports the concept of a circular economy by maximizing the use of fish by-products, which are often underutilized.

## Methods

### Chemicals

Hydrolyzed fish collagen (HFC) is from ACEF Spa (Fiorenzuola D’arda, PC, Italy) kindly supplied by Ardes s.r.l. (Sarissola, Genova, Italy). HFC derives from Tilapia and Pangasius skin. The molecular weight of HFC ranges from 1 to 3 kDa (average 2 kDa) with a protein percentage of 90%.

Acetic acid, MTT (3-(4,5-dimethylthiazol-2-yl)-2,5-diphenyltetrazolium bromide), Trizol reagent, Crystal violet (cod. 61135), chloridric acid, chloroform, and 2-propanol have been supplied by Sigma-Aldrich Chemical Company (Steinheim, Germany). Dulbecco’s Modified Eagle’s Medium (DMEM, high glucose w/l-glutamine w/sodium pyruvate), fetal bovine serum (FBS), L-Glutamine, trypsin, and Dulbecco’s Phosphate Buffered Saline w/o calcium w/o magnesium (DPBS), used for cell culture were purchased from Euroclone (Pero, Italy). The Hyp kit (perchlorate-free) from Cell Biolabs was used for the collagen quantification.

For gene expression assays, the extracted RNA was reverse transcribed using the iScript cDNA Synthesis Kit (Biorad, Milano, Italy).

### Chemical and physical properties of HFC

Stock solution of 1 mg/mL HFC (S-HFC) was prepared in 0.5 M acetic acid. The sample has been vortexed for 15 min, followed by incubation at 80 °C for 30 min. At the end, the sample has been re-vortexed for other 15 min. The UV spectrum was acquired using an Implen spectrophotometer (Quartz cuvette) after the baseline was set with 0.5 M acetic acid. The UV spectrum was collected over the wavelength range of 200–400 nm (supplementary Fig. 1 and supplementary Table 1).

The Hyp content was estimated by Hydroxyproline Assay kit (Perchlorate-Free) purchased by Cell Biolabs, Inc., (San Diego, CA, USA). Reagent preparation included incubating Chloramine T Reagent for 10–15 min at 37 °C and mixing it with Assay Buffer. For Ehrlich’s Reagent, the 2X Concentrate was warmed to room temperature and combined with diluent. Each sample (100 µL) was hydrolyzed in 12 N Hydrochloric acid at 100 °C for 3 h. Subsequently, acid-hydrolyzed samples (10 µL) were evaporated at 80 °C for 45 min. The evaporated sample was incubated with 100 µL Chloramine T Mixture (Chloramine T Reagent and Assay buffer) at room temperature (20–25 °C) for 30 min.

Then, 100 µL Ehrlich’s reagent was added to each sample and incubated for 45 min at 60 °C. After cooling to 4 °C for 5 min, followed by a centrifugation at 6000xg for 15 min at room temperature, 100 µL of the supernatant were transferred to microplate wells and the absorbance was read at 560 nm using a microplate reader (Byonoy absorbance 96, Germany). Aqueous solutions of Hyp in the concentration range of 0 to 100 µg/mL were used as standards. The calibration curve was prepared by using standard Hyp as reported in Eq. (1):

Abs560 = 0.0031 × C_Hyp_ (R^2^ = 0.9909) (1).

Where Abs560 was the sample absorbance recorded at 560 nm and **C**_**Hyp**_ was the concentration of the total concentration of Hyp (µg_Hyp_ /mL).

### Formulation of HFC

The commercial face cream (F) employed to prepare F-HFC was IALURELIX Face cream kindly supplied by Ardes s.r.l. (Sarissola, Genova, Italy). INCI: Active principle: vitis vinifera seed oil (vitis vinifera (grape) seed oil), snail secretion filtrate, sodium hyaluronate, lilium candidum bulb extract, malus domestica fruit cell culture extract, calendula officinalis flower extract. Excipient: Water, ceteareth-12, caprylic/capric triglyceride, butyrospermum parkii butter (butyrospermum parkii (shea) butter), dicaprylyl ether, carbomer, glycerin, ethylhexyl palmitate, glyceryl caprylate,, tocopherol, phenoxyethanol, tocoperyl acetate, sodium hydroxide, ethylheylglycerin, lecithin, ascorbyl palmitate, citonellol, citric acid, xanthan gum, geraniol, phonoxylglycerin, lecithin, ascorbyl palmitate, citronellol, cirtic acid, xanthan gum, geraniol, phosholipids, parfum (fragrance).

HFC was added to the cosmetic product at different concentrations (0.05%, 0.06% and 0.1% v/w, corresponding to 23.50 µg_Hyp_/mL, 28.20 µg_Hyp_/mL, and 47.00 µg_Hyp_/mL).

### Cell culture

Human Keratinocytes (HaCaT) cells were supplied by CLS Cell Lines Service GmbH (Eppelheim, Germany) and HECV by Cell Bank and Culture-GMP-IST-Genoa, Italy. Cells were cultured in Dulbecco’s modified eagle’s medium High Glucose (D-MEM) supplemented with 1% L-Glutamine and 10% Fetal Bovine Serum (FBS) at 37° C with 5% CO_2_ in humidified air.

### Preparation of S-HFC and F-HFC for cell treatments

A stock solution of S-HFC was prepared 10 mg/mL in Phosphate Buffered Saline (PBS). Cells were treated with different concentrations of S-HFC diluted in culture medium (0.1%; 0.06% and 0.05% w/v of HFC, corresponding to 47.00; 28.20; 23.50 µg_Hyp_/mL, respectively). Whereas, cells were treated with different concentrations of F and F-HFC diluted in culture medium (from 0.0001 to 0.05 mg/mL).

### Cell viability assay

15 × 103 HaCaT cells or 12 × 103 HECV cells were seeded in each well of a 96-well plate. After overnight incubation, cells were treated with different concentration of S-HFC and incubated for 24 h with 5% CO2 at 37 °C. After that cell viability was analyzed using MTT assay.

MTT was solubilized in PBS at a concentration of 5 mg/mL and filtered using 0.22 μm membrane. The working solution was diluted in culture medium at the final concentration of 0.5 mg/mL. The cells were then incubated for 3 h in a humified atmosphere with 5% CO2 at 37 °C. Formazan crystals were then solubilized in acid-alcohol (0.04 N HCl in 2-propanol) solution. The absorbance was read spectrophotometrically at 570 nm using a microplate reader (Byonoy absorbance 96, Germany)^45^.

### T-Scratch Assay on HECV cells

The T-Scratch assay is a simple method used to assess wound closure in a 2D cellular system, specifically examining cell proliferation and migration activities in response to different compounds. Notably, different concentrations of Hyp have been selected (47.00 µg/mL; 28.20 µg/mL; 23.50 µg/mL) based on their demonstrated proliferative effects in the previous cell viability assay. The protocol followed 3 days approach^46,47^.

During day one, on the back of a 12-well plate, three dots were drawn at half-centimeter intervals for each well. 150.000 HECV cells were seeded in each well using D-MEM supplemented with 10% Fetal Bovine Serum and 1% L-Glutamine. The plate was then incubated overnight at 37 °C and 5% CO_2_.

On the second day, once cells reached confluence, a 200-µL pipette tip was used to create a uniform scratch wound on the monolayer of cells. The wounded debris was removed by washing twice with PBS. Then, cells were treated with S-HFC at different concentrations of Hyp (47.00 µg/mL; 28.20 µg/mL; 23.50 µg/mL). Four images were taken for each well at 0 h (T0), precisely at the marked dots, and the plate is placed back in the incubator (37 °C, 5% CO_2_).

During the last day, four images were acquired for each well after 24 h incubation (T24).

To determine the extent of wound healing, the images were analyzed using ImageJ free software (http://imagej.nih.gov/ij/). Percentage of the closed area was measured and compared with the value obtained with CTRL. An increase of the percentage of closed area indicated the migration of cells.

### EpiDerm and EpiDerm-FT culture

The Reconstructed Human Epidermis (RHE) (EpiDerm-FT; MatTek Co., Bratislava, Slovakia) is highly differentiated 3D tissue model consisting of normal, human-derived epidermal keratinocytes (NHEK) cultured on specially prepared tissue culture inserts. The normal human 3D skin model at full thickness (EpiDerm-FT; MatTek Co., Bratislava, Slovakia) (Lot no. 34553) comprises normal human epidermal keratinocytes (NHEK) and normal human dermal fibroblasts (NHFB), cultured to form a multilayered model of the human dermis and epidermis^48^.

Upon delivery, both tissues were immediately transferred to 6-well plates and cultured in 2.5 mL DMEM (Medium EFT-400) (MatTek Co.). Tissues were restored in 5% CO_2_ at 37 °C overnight (16–18 h). At the end of the equilibration, fresh medium was added and EpiDerm and EpiDerm FT Tissues were treated topically with S-HFC and F-HFC (47.00 µg/mL; 28.20 µg/mL; 23.50 µg/mL) and then cultured at 5% CO_2_, 37 °C for 24 h.

### Skin Irritation Test on 3D EpiDerm model

Skin Irritation Test – OECD 439 was performed using 3D EpiDerm skin model (MatTek, Bratislava, Slovakia), consisting of Reconstructed Human Epidermis (RHE) with normal, human-derived epidermal keratinocytes (NHEK) cultured on specially prepared tissue culture inserts. Samples tested were prepared according to OECD 439 guidelines^32^. During the experiment, 30 µL of test substance were applied topically to the surface of the EpiDerm models and incubated at first for 35 ± 1 min in the humidified incubator (37 °C, 5% CO_2_), and then for 25 ± 1 min at room temperature. A positive control consisting in treatment with 5% SDS was included in the assays. At the end of the incubation period, the tissues were rinsed with PBS to remove any excess of test substances and then re-incubated for 24 ± 2 h at 37 °C, 5% CO_2_.

At the end of the incubation period, tissue viability was evaluated through MTT assay. Briefly, tissues were incubated with 300 µL of MTT solution (1 mg/mL) at 37 °C, 5% CO_2_. After 3 h, the excess of MTT was removed and the tissues were washed with PBS. Reduced formazan was extracted by overnight incubation with 2 mL of isopropanol. Next day, 200 µL of the extraction solution were transferred to a 96-well plate, and the Optical Density (OD) was measured spectrophotometrically at 570 nm wavelength through a microplate reader (Byonoy absorbance 96, Germany).

### Measurement of Transepithelial Electrical Resistance (TEER) on 3D EpiDerm model

TEER was performed on 3D EpiDerm immediately before MTT procedure of Skin Irritation Test. Briefly, each tissue was rinsed 2–3 times with TEER Buffer and then transferred to a 24-well plate prefilled with TEER Buffer. After an Electrode pre-equilibration (about 30 min) in TEER Buffer and addition of 300 µL of TEER Buffer on each well, measurement of TEER was performed on each EpiDerm model using an EVOM™ Epithelial Voltohmmeter (World Precision Instruments, Sarasota, FL). A positive and negative controls consisting in treatment with or without 5% SDS was included in the assays.

### Human IL-18 analysis on 3D EpiDerm model medium

The ELISA Assay for Human IL-18 detection from Abcam (Cambridge, UK) is a laboratory tool used to measure the concentration of interleukin-18 (IL-18) in biological samples, such as cell culture media. Briefly, culture media derived from EpiDerm skin models subjected to OECD 439 test and treated with S-HFC (47.00; 28.20; 23.50; µg_Hyp_ /mL) and F-HFC (47.00; 28.20; 23.50; 0.00; µg_Hyp_ /mL) were added to a 96-well plate coated with human IL-1Ra matched antibody. Then, the plate was incubated for 1 h at room temperature to allow interaction between IL-18 and the antibodies. Each well was washed to remove unbound components, and a chromogenic substrate was added for the detection of enzyme-linked antibodies. After an incubation period of 10 min, stop solution was added in each well and the intensity of the developed color was measured spectrophotometrically at 450 nm (Byonoy absorbance 96, Germany).

### RNA Extraction

After treatments, RNA was extracted from 3D EpiDerm FT tissues using Trizol reagent according to Grasselli et al.^49,50^. Briefly, 1 mL of Trizol reagent was added to each tissue. After 5 min incubation at room temperature, 200 µL of chloroform was added and every tissue was incubated for 10 min at room temperature and then centrifuged for 15 min at 15,294 x *g*, 4 °C. Three phases were formed, and the upper phase was collected and added to 500 µL of 2-propanol. After 10 min incubation at room temperature and 10 min centrifugation at 15294*g* 4 °C, the pellet was maintained and resuspended in 1 mL of Ethanol 75%, followed by 5 min centrifugation at 5974 x *g* 4 °C. Ethanol was gently removed and pellet was left to evaporate until it was dried. 50 µL H_2_O RNAse Free was added, and the total amount of RNA as well as the quality of estracted RNA of each sample was quantified using Nanophotometer NP80 (IMPLEN, Munich, Germany).

### cDNA synthesis and quantitative real-time PCR

One µg of total RNA was converted to cDNA using iScript cDNA Synthesis Kit according to the manufacturer’s instructions. Expression of the following biomarkers was evaluated: Aquaporin (AQP3) (proteins involved in the transport of water, glycerol and urea across cell membrane)^51^Collagen Type III Alpha 1 Chain (COL3A1), Collagen Type I Alpha 1 Chain (COL1A1) (both involved in the regulation of collagen expression) and Elastin (ELN) (involved in the assembly of elastic fibers) (Bio-Rad Laboratories, Hercules, CA, USA)^52^.

Quantitative RT-PCR was performed in triplicate using SYBR Green Supermix (Bio-Rad) and IQ supermix (Bio-Rad) on CFX96 Real-Time system (C1000 Thermal Cycler; Bio-Rad). The cDNA was subjected to PCR amplification cycle according to the following parameters: 95°C x 3min, 40 cycles at 95°C x 15s and 60°C x 40s. Glyceraldehyde-3-phosphate dehydrogenase (GAPDH) expression served as housekeeping gene (GAPDH Fwd: 5’- GACCCCTTCATTGACCTCAAC-3’ ; GAPDH Rev: 5’- CGCTCCTGGAAGATGGTGATGGG − 3’).

### Tissues histological analysis

EpiDerm FT tissues from the same treatments used for gene expression analysis were also fixed in 10% buffered formalin (Sigma-Aldrich, Germany), washed in PBS pH 7.4, dehydrated in ethanol, cleared in Bioclear (Bio-Optica, Italy), and paraffin embedded. They were cut at 5 μm thickness, put on slides and then stained alternatively with Hematoxylin-Eosin or Sirius red. Histological slides were observed using a light microscope Leica DMRB (Leica Microsystems, Wetzlar, Germany) equipped with cross polars and with a Moticam 3+ (Motic Europe, Spain). Sections stained with SR (Sigma-Aldrich, Germany) were used to highlight the collagen distribution within dermis, while sections stained with Hematoxylin-Eosin (Carlo Erba, Italy) were used to evaluate the number of fibroblasts within the dermis as well as dermis thickness by image analysis (ImageJ free software https://imagej.nih.gov/ij)^41^.

### Sirius Red staining for collagen – polarized light microscopy

Throughout the cross-polar light, the SR-stained histological sections show shiny green, yellow, orange collagen fibers on a dark background (see Fig. 5). For each treatment, five SR-stained sections were photographed and, in each photograph the dermis was selected in ImageJ. The function “Color threshold” was used to obtain an image with white pixels (collagen fibers) and black pixels (the background). The percentage of white pixels on the total was used as a semiquantitative evaluation of collagen presence.

### Fibroblasts count within dermis

For each treatment, five Hematoxylin-Eosin-stained histological sections were randomly chosen and photographed. In each photograph, two squares of 200 pixels x 200 pixels (0.068 mm2 in our setup) were randomly selected in the dermis. Thus, ten squares were analyzed for each treatment. Fibroblast were automatically counted in each square using ImageJ software. Briefly, the extracellular matrix was light pink-whitish, while fibroblast nuclei were purple; this allowed to use the function “Color threshold” to obtain a white background with black particles (the nuclei of the fibroblasts) to be counted using the function “Analyze Particles”. Objects smaller than 6 pixels were not considered to avoid counting artifacts due to staining procedure.

### Dermis thickness measurement

The thickness of the dermal portion of the tissues was randomly measured in five different hematoxylin-eosin-stained histological sections for each treatment. To avoid biases due to different cuts, we used the thickness of the filter as a normalization factor for sections that are not precisely orthogonal.

### Statistical analysis

Statistical analysis was performed using GraphPad Prism 10 (GraphPad Software Inc., La Jolla, CA, USA). Differences between groups were compared using one-way ANOVA using Tukey’s post hoc testing. A p value < 0.05 was considered statistically significant. Results are presented as mean ± SD.

## Electronic supplementary material

Below is the link to the electronic supplementary material.

Supplementary Material 1

## Data Availability

Data are provided within the manuscript and supplementary files; however, they can also be made available upon request. Please contact the corresponding author, Elena Grasselli (elena.grasselli@unige.it).
