# Supplementary material for: Effects of a single application of hydrolyzed fish collagen on dermal protein expression and tissue architecture in human skin models
Source: Sci Rep. 2025 Aug 11;15:29391. doi: 10.1038/s41598-025-11372-5 (PMC12340049; doi:10.1038/s41598-025-11372-5)
Supplement: Supplementary file 1 — Supplementary Material 1 [file 41598_2025_11372_MOESM1_ESM.docx]

# Supplementary Data


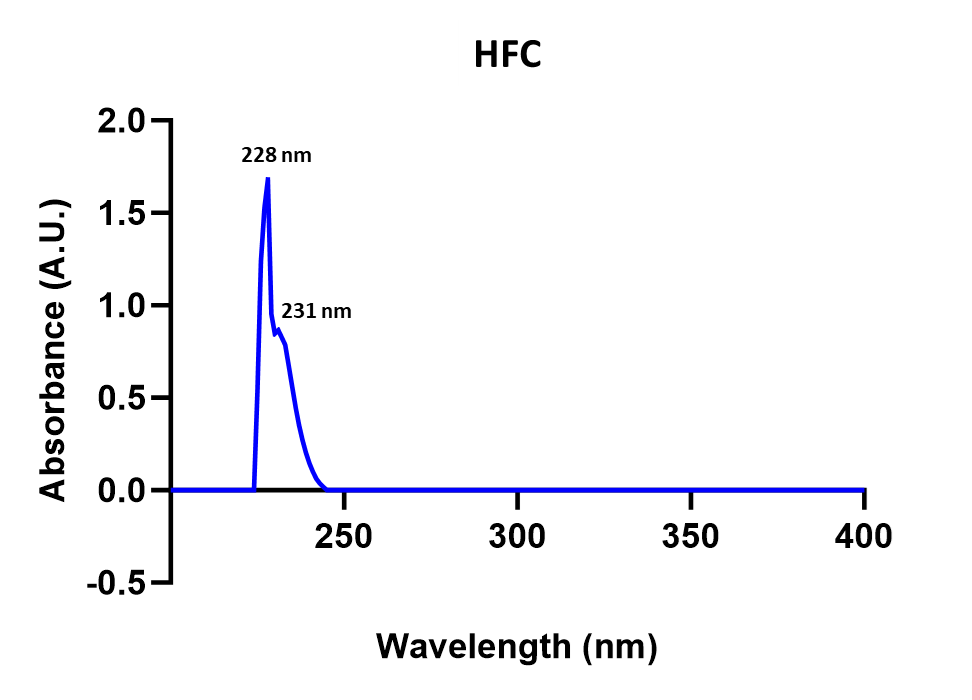


**Figure S1.** UV spectrum (ranging from 200 to 400 nm) of HFC sample at the concentration of 1 mg/mL. It was analyzed by UV-spectroscopy and it displays a prominent peak around 228 nm which can attributed to the presence of Collagen inside the sample, as demonstrated by literature ^44^.

**Table S1**. HFC – Representative characteristics of the spectrum.

| **Sample name** | **Major peak**  **Wavelength (nm)** | **Major peak**  **(Abs)** | **Minor peaks**  **Wavelength (nm)** | **Minor peaks**  **(Abs)** |
| --- | --- | --- | --- | --- |
| HFC | 228 | 1.68 | 209; 232 | 0.211; 0.825 |
